# The Role of Gut Bacteria and Plasma Metabolites in Predicting Post-Stroke Depression in Patients with Acute Ischemic Stroke

**DOI:** 10.2174/011570159X390349250818115524

**Published:** 2025-09-09

**Authors:** Lulu Wen, Tong Si, Chuming Yan, Huixin Shen, Wancheng Zheng, Meihong Xiu, Miao Qu

**Affiliations:** 1 Neurology Department, Xuan Wu Hospital Capital Medical University, Beijing, China;; 2 Neurology Department, Beijing Shijitan Hospital, Beijing, China;; 3 Beijing Huilongguan Hospital, Peking University Huilongguan Clinical Medical School, Beijing, China

**Keywords:** Post-stroke depression, gut bacteria, metabolites, biomarkers, prediction, Acute Ischemic Stroke (AIS)

## Abstract

**Introduction:**

Early diagnosis of Post-Stroke Depression (PSD) is challenging. This study aimed to identify possible biomarkers in gut microbiota and plasma metabolites within 72 hours after Acute Ischemic Stroke (AIS) to predict PSD occurring 2 weeks later.

**Method:**

In this study, 86 patients with AIS were observed within 3 days of stroke onset and followed up for 2 weeks. We collected the feces and plasma within 72 hours of AIS onset for 16S rRNA sequencing and liquid chromatography-mass spectrometry analysis, respectively.

**Results:**

At the genus level, PSD patients at 2 weeks following a stroke had a higher relative abundance of *Blautia*, *Eubacterium_hallii_group*, *Tyzzerella,* and a lower abundance of *Ellin6067*, *Massilia*, *Luedemannella,* and *Gemmataceae_others *within 3 days of AIS onset. Meanwhile, when all metabolites in plasma collected within 72 hours after AIS onset were used to predict 2-week PSD, 31 altered metabolites were identified, of which 28 metabolites increased and 3 decreased, belonging predominantly to steroid and steroid derivatives, glycerophospholipids, fatty acyls, and prenol lipids. The Area Under the Curve (AUC) values for the clinical data, metabolic profiles, gut microbiota, and combined dataset were 0.664 (0.549,0.779), 0.739 (0.621, 0.857), 0.870 (0.781,0.960), and 0.955 (0.888,1), respectively.

**Discussion:**

Our study identified potential biomarkers from clinical data, gut bacteria, and plasma metabolites that contribute to PSD. Within 72 hours after AIS, combining these biomarkers from all three sources showed preliminary ability to predict PSD at 2 weeks. Metabolites had the highest contribution, followed by gut bacteria and clinical data.

**Conclusion:**

A biomarker panel including metabolites, gut microbiota, and clinical data within 72 hours after AIS onset could preliminarily predict PSD 2 weeks later.

## 1 INTRODUCTION

Post-Stroke Depression (PSD) is a general neuropsychiatric disorder in patients with stroke. Studies have shown that 11% to 41% of stroke survivors develop secondary depressive symptoms between 2 weeks and 2 years after stroke, a large and noteworthy population [1]. In addition, PSD negatively affects the rehabilitation of stroke patients and is related to reduced independence in daily activities and an increased risk of all-cause mortality [2, 3]. Therefore, early identification and intervention of PSD after Acute Ischemic Stroke (AIS) are essential to improve the prognosis of PSD patients. However, PSD often faces insufficient diagnosis and treatment due to a lack of available biomarkers. In particular, stroke patients usually leave the hospital within about 2 weeks and go to the outpatient clinic for further consultation at 3 months after the onset of the stroke [4]. Therefore, exploring potential biomarkers within 72 hours of AIS onset to predict PSD after 2 weeks could help physicians make early intervention decisions and avoid diagnostic and therapeutic delays in PSD.

Evidence indicates that disruption of the gut microbiota may play a critical role in the pathogenesis of PSD. Previous literature has reported that animal models of PSD had altered species richness indices and significantly disturbed microflora composition [5, 6]. Fecal microbiota transplantation ameliorated the disturbed microflora structure and increased the monoamine neurotransmitters in the brain, which ameliorated depression in PSD rats [7, 8]. A recent meta-analysis showed significant changes in microbial community structure at the phylum, family, and genus levels in patients with PSD compared with healthy controls [9]. To our best knowledge, the differences in gut microflora composition in stroke patients with and without PSD have not been fully explored, which is important to elucidate the pathogenesis and predict the occurrence of PSD.

Metabolomics focuses on metabolite profiling in organisms and is routinely applied to discover biologically important metabolic pathways in disease states. Accumulating studies have identified various plasma metabolites [10-13] or urinary metabolites [14-16] that can serve as biomarkers of PSD, mainly related to amino acid metabolism, lipid metabolism, *etc*. Both animal and clinical studies have shown that lipid metabolism may be associated with the development of PSD [7, 17]. Notably, plasma metabolomics can reflect organismal metabolic changes more directly than urine metabolomics. Peripherally circulating metabolites may be affected by the gut microbiota, which then transmit signals to the brain [18]. Therefore, combined gut microbiota and metabolomics may be better to explore the mechanism of PSD or to predict PSD.

The present study was designed to jointly determine the gut microbiome and plasma metabolomics of patients with AIS by collecting clinical data, feces, and plasma within 72 hours after onset and following up at 2 weeks to identify early potential biomarkers of PSD.

## 2 MATERIALS AND METHODS

### 2.1 Participants

This prospective cohort study was registered in clinical trials (ChiCTR2100041895) and was carried out from June 2021 to April 2022 in a university hospital. The protocol was approved by the Ethics Committee of Xuanwu Hospital of Capital Medical University (LYS [20^20^]096). One hundred and three participants with AIS were admitted to the ward within 72 hours after the onset of AIS, and 86 patients were followed up in the outpatient clinic for 2 weeks, with a dropout rate of 16.5%. In addition, written informed consent was obtained from each patient. In particular, for patients with cognitive impairment, the guardian signed an informed consent form.

The inclusion criteria at baseline (72 hours after AIS onset) included at least 18 years of age, diagnosis of AIS by MRI within 72 hours after AIS onset, and clear consciousness, as well as willingness to cooperate. The exclusion criteria included a history of depression or other mental disorders and serious systemic diseases such as cancer, unstable vital signs, cerebral hemorrhage, exposure to antibiotics within 3 months or probiotics and prebiotics within 2 weeks, and a history of severe intestinal diseases.

Participants recruited at baseline were further interviewed at 2 weeks by the experienced clinicians. A final diagnosis of PSD was made using DSM-5 criteria for depression. The severity of depressive symptoms was assessed by Hamilton Depression Rating Scale (HAMD) scores. Participants with HAMD scores≥ 8 and diagnosed with depression were categorized into the PSD group.

All clinical data were obtained from the inpatient electronic medical record system, including demographic information, medical history, empirical results, imaging findings, and NIHSS scores.

### 2.2 16S rRNA Sequencing and Untargeted Metabolome Analysis

DNA from fecal samples was extracted as previously described [19]. The 16S rRNA gene was amplified, and the Illumina TruSeq DNA PCR-Free Library preparation kit (Illumina, USA) was used to generate a sequencing library. The library was sequenced on the Illumina Nova Seq platform to generate 250 bp double-ended reads. Sequences were analyzed using the QIIME software package. The linear discriminant analysis effect size (LEfSe) was used to screen the discriminant classification units of intestinal bacteria with significant differences between the PSD group and the non-PSD group.

A100 μl plasma from each sample was used for untargeted metabolome analysis. The detailed procedure of RNA sequencing and untargeted metabolome analysis was described in Supplementary File **1**.

### 2.3 Statistical Analysis

According to the normality test, the continuous variable was described as mean ± SD or median and quartile (Q1, Q3). Student t-tests or nonparametric Mann-Whitney U tests were used to compare continuous variables between groups. The categorical variable was presented as n (%) and calculated by the X^2^ test. Fisher’s exact test was used to determine differences between groups. Statistical analyses were performed using SPSS 26.0 (SPSS, Inc., Chicago, IL). Statistical difference was considered when a two-sided *P*-value < 0.05.

For metabonomic analysis, log transformation was used to make it close to a normal distribution, and z-score was used to balance the variance of different metabolites. Metabolites with a high missing rate (>50% of samples missing) were deleted, and the multiple imputation method was used to fill in the missing values. Total Ionic Current (TIC) normalization was used to eliminate the systematic errors between samples. Principal Component Analysis (PCA) score plots and OPLS-DA analysis were carried out using MetaboAnalyst 5.0. Metabolite with VIP>1 and *P*-value <0.05 was confirmed as a statistical difference. Spearman’s rank correlation was performed to explore the associations of significant bacteria and metabolites with clinical data. In each dataset, logistic regression analyses were used to screen for characteristic variables based on different variables, and there is no multicollinearity. Based on the characteristic variables, PLS-DA was carried out using the “mixOmics” R package to distinguish sample differences between groups. Receiver Operating Characteristic curves (ROC) were further carried out using the “pROC” packages in R (R Foundation; http://www.r-project.org; version 4.1.0).

## 3 RESULT

### 3.1 Subject Information at Baseline

Eighty-six patients with AIS were recruited after 72 hours of stroke onset. The demographic characteristics, medical history, laboratory data, and neuropsychological function of patients based on the diagnosis of PSD are listed in Table **1**. Patients were followed up at week 2. At this follow-up, 37(43.02%) patients were diagnosed with PSD. PSD patients had higher HAMD scores at the 2-week follow-up (*P*<0.05). No significant differences were observed in age and gender between PSD and non-PSD patients (all *P*>0.05). There were no significant differences in hypertension, diabetes, coronary heart disease, and hyperlipidemia between the two groups (all *P*>0.05). In terms of laboratory data at baseline, PSD patients had elevated Prothrombin Time (PT), decreased Mean Platelet Volume (MPV), and prealbumin levels than non-PSD patients (all *P*<0.05). There was no difference in Platelet Distribution Width (PDW), Alanine Aminotransferase (ALT), Glutamyl Transpeptidase (GT), High-Density Lipoprotein (HDL), Low-Density Lipoprotein (LDL), uric acid, and Apolipoprotein B (apoB) between the PSD and non-PSD groups. Besides, no significant differences were shown in infarction location, MoCA, and NHISS score between the two groups (all *P*>0.05). However, the proportion of large-artery atherosclerosis of the TOAST type was higher in non-PSD patients (*P*<0.05).

### 3.2 Baseline Gut Bacteria for Predicting 2-Week PSD

To determine whether the composition of gut bacteria within 72 hours after AIS correlated with PSD at 2 weeks, fecal samples were collected and tested by 16S RNA sequencing. As shown in Fig. (**1**), β-diversity was calculated, and weighted UniFrac PCoA showed no difference in baseline gut bacteria composition between the two groups (Fig. **1A**, *P*>0.05). In addition, no difference was observed in α-diversity values between baseline samples from PSD and non-PSD patients, including the species diversity indices (Shannon and Simpson) and the species richness indices (Ace and Chao) (Figs. **1B**-**E**, *P*>0.05). LEfSe analysis and Wilcoxon rank-sum test were performed to identify differential bacterial taxa between PSD and non-PSD patients at baseline (LDA score>2, and *P*<0.05, Figs. **2A**, **B**). Differential bacteria at baseline included decreased *Epsilonbacteraeota* at the level of phylum and decreased *Planctomycetacia* at the level of class in the non-PSD group. However, at the level of order, the PSD group had an increase in *Enterobacteriales* and a decrease in *Micromonosparales* and *Gemmatales*. At the level of family, enhanced *Enterobacteriaceae* and decreased *Sporichthyceae*, *Micromonosporales,* and *Gemmataceae* were found in the PSD group. Furthermore, at the level of genus, PSD patients had a higher abundance of *Blautia*, *Eubacterium_hallii_group*, *Tyzzerella,* and a lower abundance of *Ellin6067*, *Massilia*, *Luedemannella,* and *Gemmataceae_others* at baseline.

### 3.3 Disturbances of Baseline Plasma Metabolites for Predicting PSD at 2 Weeks

To determine whether plasma metabolites within 72 hours after AIS were associated with PSD at 2 weeks, untargeted metabolomic analysis was carried out. Considering the possible association between gut microbiota and plasma metabolites, LC-MS based on untargeted metabolomic analysis was performed. PCA is an unsupervised analysis method that initially reflects the overall differences between groups and the variability between samples within the group, while OPLS-DA is a supervised discriminant analysis method, which can weaken the intra-group differences and make the inter-group sample separation effect better [20]. PCA and OPLS-DA models showed significant differences in baseline plasma metabolic profile between PSD and non-PSD patients (Figs. **3A**, **B**). Compared with non-PSD patients, 31 plasma metabolites were altered at baseline in patients diagnosed with PSD at 2 weeks, with 28 metabolites increased and 3 metabolites decreased (Table **S1**). Specifically, seven metabolites in patients with PSD were steroid and steroid derivatives, including elevated progesterone, dolichosterone, secasterone, chenodeoxycholic acid, 5a-tetrahydrocorticosterone, and cholic acid, and decreased cholesterol sulfate. Four metabolites were glycerophospholipids, including increased PC(P-18:1(9Z)/ 18:0), PC(16:1(9Z)/P-18:1(11Z)) and PC(18:1(9Z)/P-18:1 (11Z)), and decreased PC(22:2(13Z,16Z)/14:1(9Z)) in PSD patients. Three metabolites were fatty acyls, including increased 13S-hydroxyoctadecadienoic acid, decanoylcarnitine, and caprylic acid in PSD patients. Five elevated metabolites in PSD patients were all prenol lipids, including barringtogenol C, ganoderiol C, 22-acetylpriverogenin B, camelliagenin A, and trimethylamine N-oxide (TMAO). Finally, eleven other metabolites increased in the baseline plasma of PSD patients, including 2-Ethyl-3,5-dimethylpyrazine, 2-Methylpiperidine, 3-(4-Hydroxyphenyl)-1-propanol, 4-Aminobutyraldehyde, 1,1'-[1] bisbenzene, 2,2,6,7-Tetra-methylbicyclo[4.3.0]nona-1(9),4-diene-7,8-diol, DG(15:0/18:3 (6Z,9Z,12Z)/0:0), glycolic acid, 3-Methoxy-4-hydroxyphenyl-glycol sulfate, alpha-N-Phenylacetyl-L-glutamine and [12]-Gingerol. However, 1-nitrosonaphthalene was decreased in plasma from PSD patients at baseline (Fig. **3C**).

### 3.4 Correlation between Metabolites and Gut Bacteria

Correlation analysis found that PC (18:1(9Z)/P-18:1 (11Z)) was positively associated with *Blautia* and negatively associated with *Luedemannella* and *Ellin6067*. Ganoderiol C was inversely associated with *Ellin6067*, while DG (15:0/ 18:3(6Z,9Z,12Z)/0:0) was significantly correlated with *Eubacterium_hallii_group* (Fig. **4**). In addition, PC (22:2(13Z,16Z)/14:1(9Z)) and Luedemannella, 1-nitrosona-phthalene, and *Massilia* all had a direct positive correlation, while cholesterol sulfate was inversely correlated with *Gemmataceae and others*. These data revealed a close association between altered metabolites and gut bacteria.

### 3.5 Modeling of Clinical Features, Gut Bacteria, and Metabolites Datasets for Predicting PSD

We performed PLS-DA analysis based on significant features of clinical data, gut bacteria, and metabolites. As shown in Fig. (**5A**), the main contributors distinguishing PSD from non-PSD patients were those significant metabolites, followed by gut bacteria and the clinical features mentioned above.

In the clinical data, peripheral blood MPV and TOAST type were key factors, whereas potentially significant biomarkers in the metabolite dataset were decanoylcarnitine, camelliagenin A, 1-nitrosonaphthalene, and 4-amino-butyraldehyde. All seven important gut bacteria were included in the gut bacteria dataset. Three subsets of clinical data, gut bacteria, and metabolites were further integrated into the combination dataset.

The AUCs for clinical data, gut bacteria, and metabolomics within 72 hours after AIS were 0.664 (0.549, 0.779), 0.739 (0.621, 0.857), and 0.870 (0.781, 0.960), respectively (Fig. **5B**). Moreover, the combined dataset predicted PSD with an AUC value of 0.955 (0.888, 1) at 2 weeks.

## 4 DISCUSSION

We analyzed the difference in clinical data, gut bacteria, and plasma metabolites between the two groups. Multi-omics analysis revealed an important role of altered gut bacteria and plasma metabolites in the development of PSD. More importantly, we identified a panel of gut bacteria, plasma metabolites, and laboratory tests within 72 hours after AIS onset to preliminarily predict 2-week PSD.

Gut microbiome disorders have been reported to be strongly related to the central nervous system, including stroke and depression [21-23]. Consistent with prior literature, we demonstrated the critical role of the gut microbiome in the development of PSD. For example, a recent study has found that when predicting 3-month PSD patients with a gut microbiome within 72 hours after stroke onset, there was a significant increase in the genus *Streptococcus*, *Akkermansia*, and *Barnesiella*, and a significant decrease in the genus *Escherichia-Shigella*, *Butyricicoccus*, and *Holdemanella* in PSD patients as compared with non-PSD patients [24]. Our study showed that at the level of genus, a higher abundance of *Blautia*, *Eubacterium_hallii_group*, *Tyzzerella*, and a lower abundance of *Ellin6067*, *Massilia*, *Luedemannella,* and *Gemmataceae_others* within 72 hours were able to predict PSD 2 weeks after AIS onset. The differential bacteria in these two studies may be due to different patient populations and follow-up time points (2 weeks *vs*. 3 months). In addition, *Blautia* is a genus of anaerobic bacteria with potential probiotic characteristics that have been associated with a variety of human diseases [25]. Previous studies have suggested that treatment with the *Blautia* genus significantly ameliorates obesity, type 2 diabetes in humans, and social deficits in autism mouse models [26, 27]. However, a higher abundance of *Blautia* in fecal microbiota has been reported in irritable bowel syndrome, ulcerative colitis, and early-stage breast cancer with deteriorating clinical stage and histoprognostic grade [28-30]. These conflicting studies have revealed that different species of *Blautia* may play beneficial or harmful roles in various human diseases and that more concise assays by metagenomic sequencing and animal experimental verification of individual strains are necessary to elucidate the function of bacteria at the species level.

Besides, Mendelian genetic analysis showed a causal relationship between *Enterobacteriaceae* and schizophrenia, and animal experiments also showed a significant increase of *Enterobacteriaceae* in Parkinson's disease mice [31, 32]. Recent studies have shown that periodontitis salivary microbiota exacerbates colitis-induced anxiety-like behavior *via* gut microbiota, and oral microbiome dysbiosis is a risk factor for stroke, both of which indicate the important role of the oral-gut-brain axis in the central nervous system [33, 34]. Interestingly, the salivary microbiome of children with dental caries showed increased *Enterobacteriaceae*, while a Mendelian randomization study strengthens the bidirectional connection between *Enterobacteriaceae* and periodontitis [35, 36]. Therefore, whether *Enterobacteriaceae* can lead to PSD *via* the oral-gut-brain axis should be further verified.

*Tyzzerella* is a genus of gram-negative, spore-forming *Bacillus piliformis* [37]. The high relative abundance of *Tyzzerella* was found in postpartum depressive disorder, progressive multiple sclerosis, and postherpetic neuralgia [38-40]. Interestingly, one study has shown that beneficial bacteria can alleviate high-fat-diet-related metabolic-associated fatty liver disease *via* decreasing *Tyzzerella,* and another clinical trial showed that probiotics can reduce cognitive impairment related to bacteria such as *Tyzzerella* and improve cognitive functions in healthy elderly people [41, 42]. In addition, animal studies have shown that *Tyzzerella* is a key bacterium for depressive disorders in mice, which is related to 5-HT, 5-hydroxyindoleacetic acid levels, and glutamic acid levels. Antidepressants can significantly reduce the abundance of Tyzzerella in depressed mice [43]. Thus, *Tyzzerella* may contribute to the pathogenesis of post-stroke depression, whose specific function remains to be further studied.

In the non-targeted metabolomics in our study, the differences in metabolite profiles, particularly lipid metabolism profiles within 72 hours after AIS onset, were important in predicting PSD at 2 weeks after onset. In partial agreement with the results of the animal model of PSD, the 4 types of Phosphatidylcholines (PCs) were significantly changed in the PSD group compared with the stroke group [17]. We observed an increase in 3 types of PCs and a decrease in 1 type of PC in the plasma of patients diagnosed with PSD at 2 weeks after AIS onset, compared to patients without PSD. PC is a key component of cell membranes and is derived mainly from dietary intake and biosynthesis in organisms [44]. PCs have anti-oxidants and anti-inflammation properties, and PC supplementation may improve depressive-like behavior and dementia [45-47]. However, one study suggested that only EPA/DHA-PC derived from sthenototeuthis oualaniensis prevented insulin resistance, whereas soybean PC and egg yolk PC did not [48]. Meanwhile, another study has also shown that PC supplementation can attenuate vaccine-induced depressive-like behavior in mice [49]. Besides, several types of PCs were increased in the mouse model of anxiety, and pharmacological treatment further increased these PCs and significantly decreased other types of PCs [50]. These studies have suggested that PCs play an important role in the development of human diseases. Particularly, an integrated meta-analysis showed that lower peripheral blood phosphatidylcholine (32:1) was associated with Major Depressive Disorder (MDD) [51], and another animal study has proven that 1,2-dilinoleoyl-sn-glycero-3-phosphocholine (DL-PC) and 1-palmitoyl-2-oleoyl-sn-glycero-3-phosphocholine (PO-PC) could ameliorate stress-induced depression-related behaviors [52]. Few studies have explored the role of PCs in PSD patients, and a cohort study showed that 17 PCs, such as PC (16:1(9Z)/14:0), were significantly reduced in PSD patients, which is different from MDD [53]. Besides, Blautia was positively correlated with PC(18:1(9Z)/P-18:1(11Z)); the mechanism of Blautia and specific PC in PSD remains to be further investigated.

Additionally, in plasma collected within 72 hours after AIS onset, we also found increased Trimethylamine N-Oxide (TMAO) in patients diagnosed with PSD at 2 weeks compared with non-PSD patients. Interestingly, the gut microbiota can metabolize dietary phosphatidylcholines to produce TMAO, which is positively associated with ischemic brain lesions after carotid artery stenting and dementia [54, 55]. A recent study has shown that TMAO can enhance post-stroke depression progression *via* ROS-p38/MAPK signaling [56]. This reveals that TMAO exerts a deleterious effect on the pathogenesis of PSD.

Carnitine is an important nutrient derived from the diet or endogenously synthesized [57, 58]. Recent studies have shown that acylcarnitine plays an essential role in the catabolism of fatty acids through mitochondrial β-oxidation, which is closely related to energy supply, ketone body production, and cell homeostasis [59-61]. Some studies have shown that acylcarnitine may be associated with increased insulin resistance and oxidative stress [62, 63]. In our study, decanoylcarnitine levels were increased in PSD patients. Previous studies have shown that decanoylcarnitine is one of the independent discriminants for stroke recurrence [64]. Another study showed that decanoylcarnitine is markedly elevated in the aqueous humor of glaucoma patients [65]. However, decanoylcarnitine can improve liver mitochondrial dysfunction by enhancing fatty acid β-oxidation [66]. Therefore, whether decanoylcarnitine exerts a positive effect on PSD should be further verified in the future.

Diagnosis or predictive tools with more objective indicators of PSD are necessary at present [67]. In our study, a series of differential features has been shown to contribute to the pathogenesis of PSD. We established a classification model by screening potential biomarkers from clinical data, gut bacteria, and plasma metabolites. Notably, although PSD could be exactly predicted from several potential biomarkers in each dataset, the combination of biomarkers from three datasets within 72 hours after AIS showed preliminary ability to predict 2-week PSD. Metabolites contributed the most to the combination prediction, followed by gut bacteria and clinical data.

 However, this is a preliminary study. A multi-center validation study is warranted to enhance the generalizability and robustness of the predictive model. The study will include a large sample of patients with and without PSD. In addition, data will be randomly allocated into internal training and validation cohorts. To ensure geographical and clinical diversity, external validation cohorts will be established at four major hospitals across different regions of China. This approach aims to capture the diverse patient demographics, regional healthcare practices, and potential confounding factors, thereby enhancing the model’s real-world applicability [68].

## STUDY LIMITATIONS

Firstly, the sample size was limited, external validation is lacking, and large sample multi-center research should be conducted in the future to generalize our research results. Causative links between gut microbiota and plasma metabolites should be verified in cohort studies or animal experiments. Secondly, in the future follow-up study, stroke patients with a history of depression can be included for subgroup analysis. Besides, diet and lifestyle affect gut microbiota and metabolites, which should also be considered in future studies [69, 70]. Lastly, key metabolites (*e.g*., TMAO, PCs) require targeted metabolomics assays for quantification, and their specificity to PSD should be verified in further mechanism studies. Importantly, Longer-term follow-up is crucial for patients with PSD. We will continue to focus on long-term follow-up and data collection with a larger sample size in our future work. Despite these limitations, this study first integrated clinical data, gut bacteria, and plasma metabolites within 72 hours after AIS to predict 2-week PSD and to reveal possible mechanisms of PSD. Especially, the combination of an early sampling window (within 72 hours), a 2-week predictive endpoint, and integrated modeling together represents a novel methodological contribution that complements, rather than duplicates, prior work.

## CONCLUSION

This study first integrated multi-dimensional data within 72 hours after AIS onset to predict 2-week PSD preliminarily. Gut bacteria such as *Blautia* and *Enterobacteriaceae* may contribute to the pathogenesis of PSD, and plasma metabolites such as PCs and TMAO may also participate in the occurrence of PSD. Within 72 hours after AIS, a panel of combined biomarkers from metabolites, gut bacteria, and clinical data was demonstrated for early predicting 2-week PSD, which may help facilitate early diagnosis and treatment of PSD.

## Figures and Tables

**Fig. (1):**
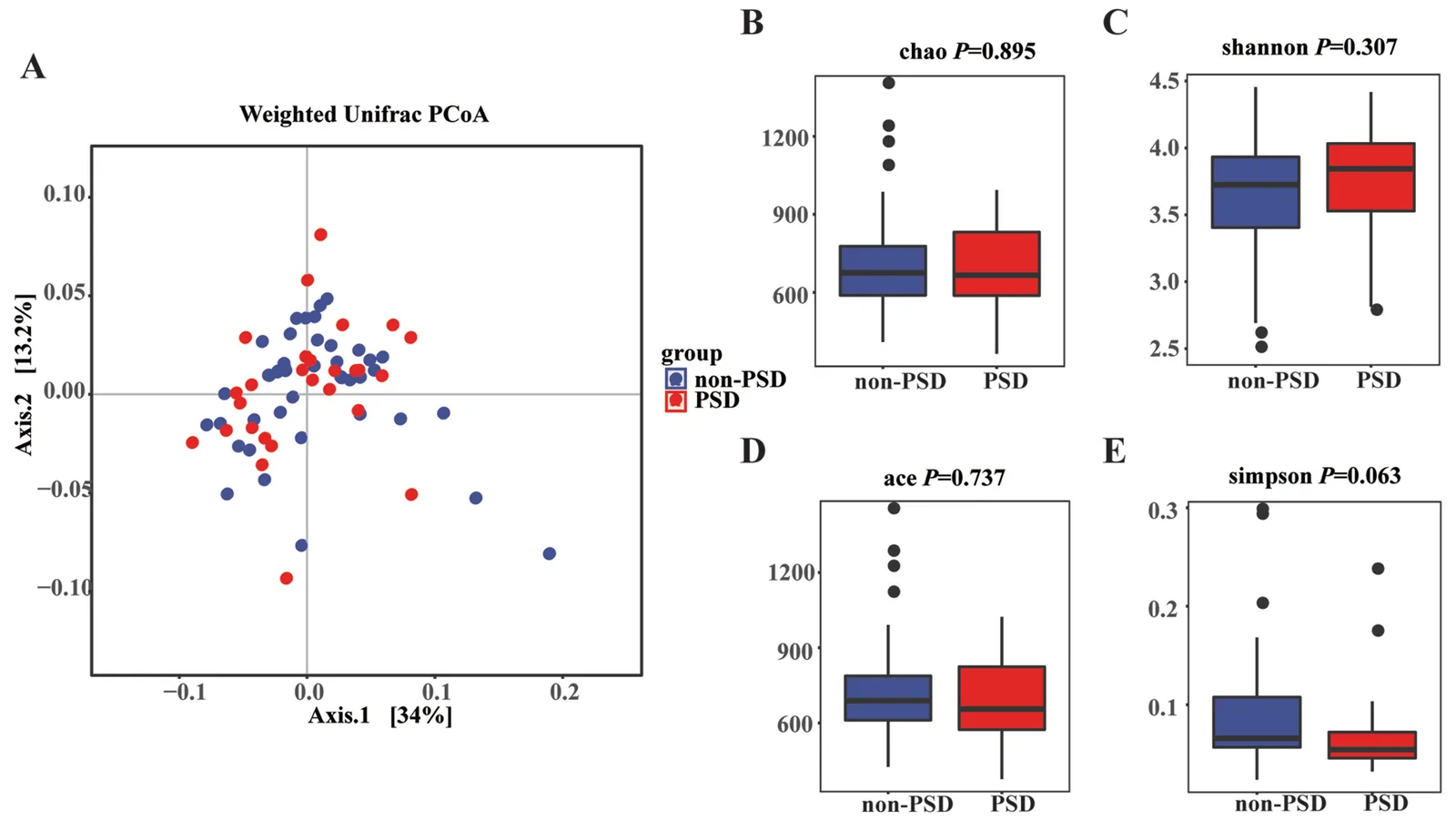
Diversity and structure of gut microbiota in PSD and non-PSD patients. (**A**) Weighted unifrac PCoA showed β-diversity between two groups. (**B**-**E**) α-diversity represented by species richness indices (Ace (**B**) and Chao( **D**)) and species diversity indices (Shannon( **C**) and Simpson(**E**)).

**Fig. (2):**
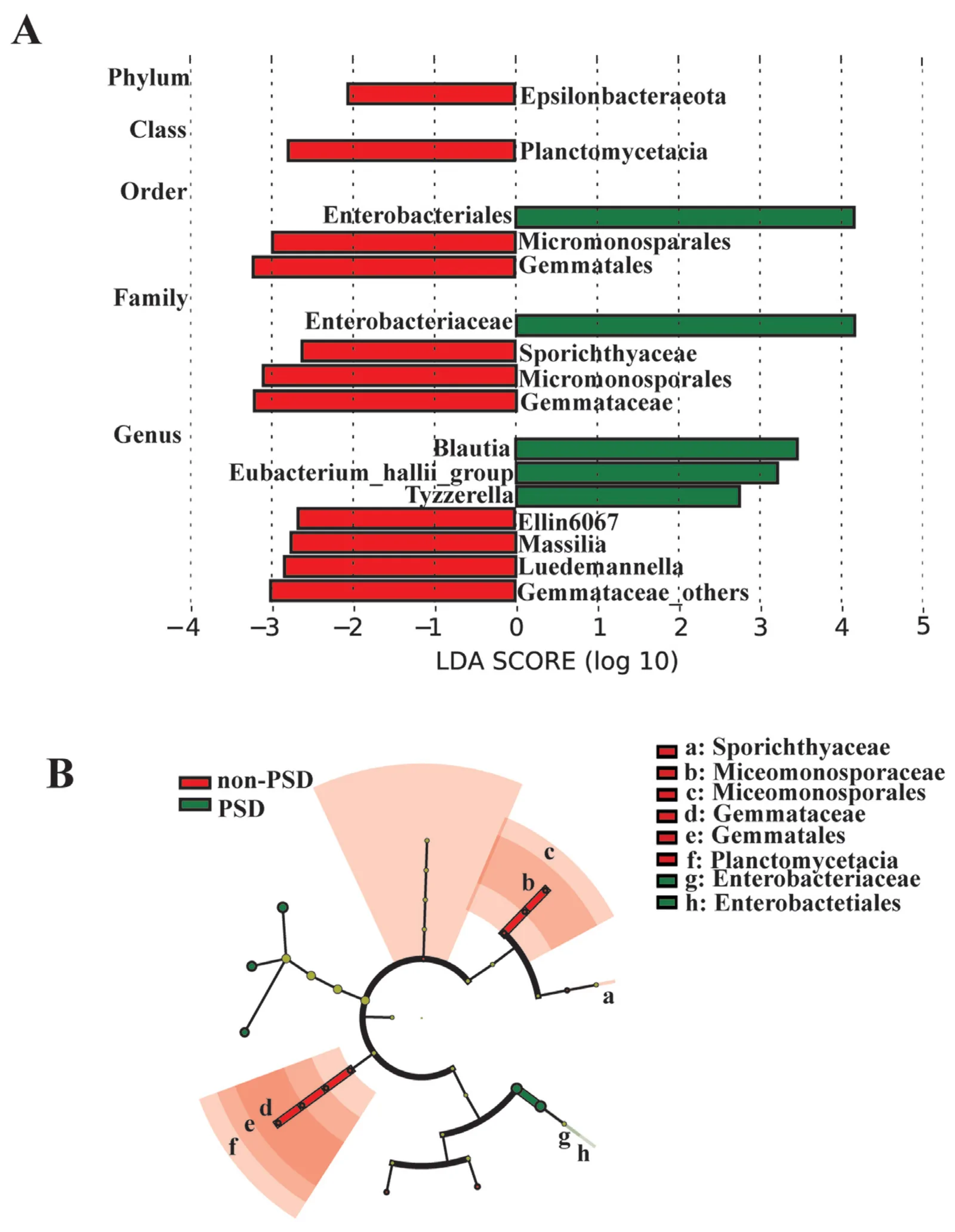
Linear discriminant analysis integrated with effect size (LEfSe) analysis. (**A**, **B**) Cladogram (**A**) and histogram (**B**) illustrated the differential gut bacteria for discriminating PSD and non-PSD patients.

**Fig. (3):**
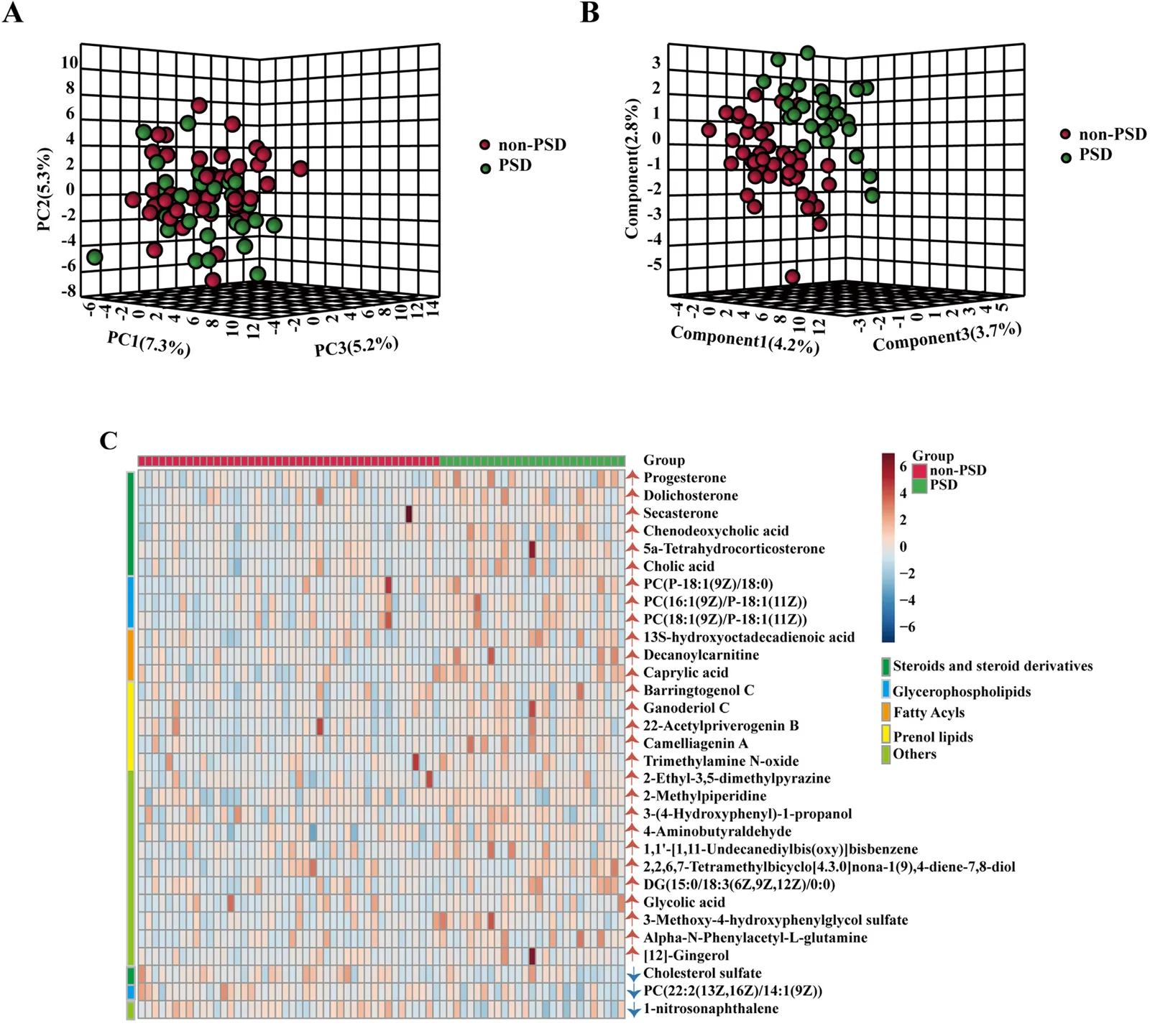
Plasma metabolism characteristics at baseline for predicting 2-week PSD. (**A**) PCA showed significant differences in plasma metabolites between PSD and non-PSD patients. (**B**) OPLS-DA excluded intra-group difference and further showed significant differences in plasma metabolites between PSD and non-PSD patients. (**C**) 31 differential metabolites for predicting 2-week PSD showed by heatmap.

**Fig. (4):**
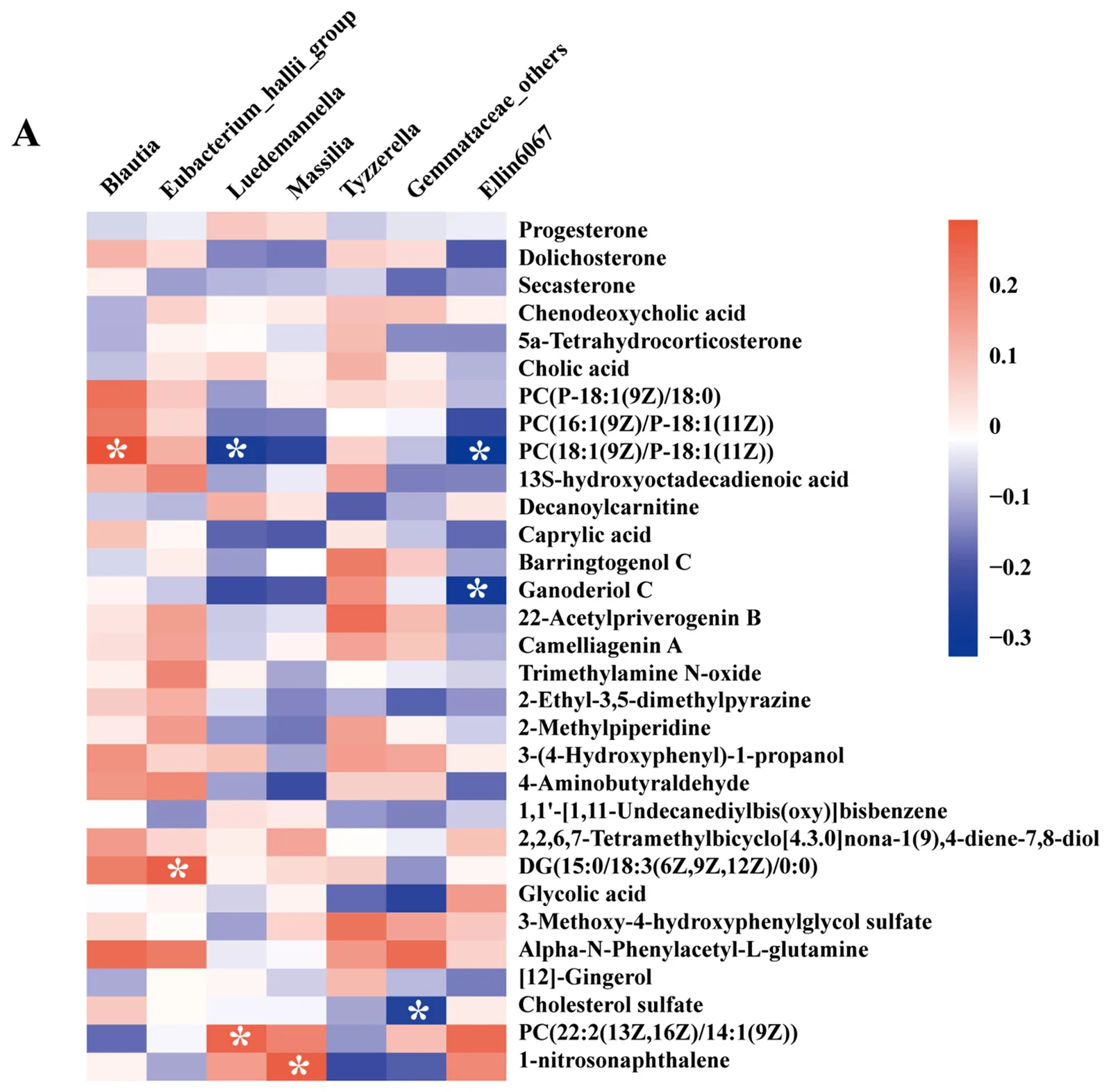
Associations between altered gut microbes with plasma metabolites. (**A**) Heatmap of Spearman’s rank correlation of 31 differential metabolites and 6 discriminative gut bacteria at the genus level. The pink rectangle indicates a positive correlation, while the blue rectangle indicates a negative correlation (*p < 0.05).

**Fig. (5):**
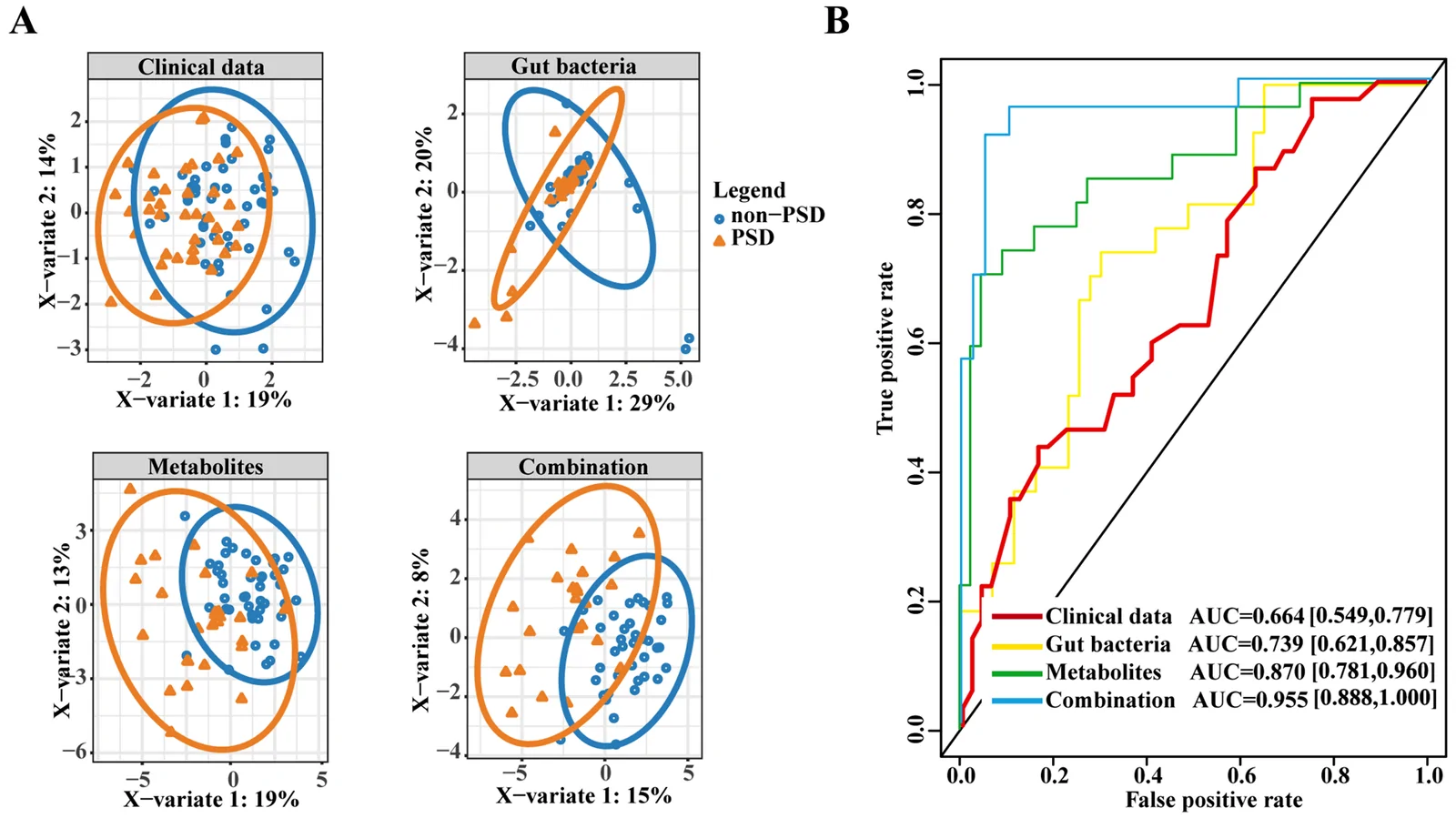
Modeling of clinical data, gut bacteria, and metabolite datasets. (**A**) PLS-DA consensus plot for clinical data, gut bacteria, metabolites, and combination datasets. (**B**) The ROC curves with area under the curve (AUC) values represented the predictive ability in each dataset.

**Table 1:** Clinical variables assessed at admission.

**Variables**	**Non-PSD (n=49)**	**PSD(n=37)**	***P*-value**
Age	60(50-66)	62(56.5-71)	0.565
Sex	38(77.6)	25(67.6)	0.300
Hypertension	32(65.3)	25(67.6)	0.826
Diabetes	14(51.9)	13(48.1)	0.516
CHD	9(18.4)	8(21.6)	0.708
Hyperlipidemia	26(53.1)	24(64.9)	0.272
PT(s)	12.7(12.2-12.9)	12.8(12.25-13.1)	0.044*
PDW(CV%)	12.4(11.1-14.5)	11.8(10.9-12.6)	0.087
ALT (U/L)	16.0(13.0-25.0)	14.0(11.0-17.5)	0.057
GT(U/L)	25.0(20.0-35.0)	22.0(16.5-28.5)	0.070
MPV (fL)	10.70 ± 0.90	10.30 ± 0.66	0.009*
Prealbumin (mg/L)	255.2 ± 51.34	236.43 ± 49.14	0.043*
UA(umol/L)	337.88 ± 83.18	303.97 ± 67.19	0.239
HDL(mmol/L)	1.03 ± 0.24	1.15 ± 0.30	0.291
LDL(mmol/L)	2.87 ± 0.94	2.57 ± 0.70	0.138
apoB(g/L)	0.98 ± 0.23	0.87 ± 0.17	0.153
NIHSS score	2.92 ± 3.59	4.30 ± 4.16	0.103
Infarction location	-	-	0.619
Frontotemporal lobar	4(8.2%)	2(5.4%)	-
Other area	45(91.8%)	35(94.6%)	-
MoCA	24.51 ± 3.31	23.78 ± 3.55	0.331
TOAST type	-	-	0.021*
LAA type	38 (77.6)	20(54.1)	-
Other type	11(22.4)	17(45.9)	-
HAMD score	5(2-7)	13.5(7-18.5)	<0.001*

**Abbreviations**: CHD: Coronary Heart Disease; PT: Prothrombin Time; PDW: Platelet Distribution Width; MPV: Mean Platelet Volume; ALT:Alanine aminotransferase; GT: Glutamyl Transpeptidase; UA:Uric Acid; HDL:High Density Lipoprotein; LDL: Low Density Lipoprotein;apoB:apolipoprotein B; NIHSS, National Institutes of Health Stroke Scale; MoCA: Montreal Cognitive Assessment; TOAST:Trial of Org 10172 in Acute Stroke Treatment; LAA type:Large-Artery Atherosclerosis type. * represents *P*<0.05.

## Data Availability

The data and supportive information are available within the article.

## LIST OF ABBREVIATIONS

AIS: Acute Ischemic Stroke
ALT: Alanine Aminotransferase
ApoB: Apolipoprotein B
DL-PC: 1,2-dilinoleoyl-sn-glycero-3-phosphocho-line
DSM-5: Diagnostic and Statistical Manual of Mental Disorders, Fifth Edition
GT: Glutamyl Transpeptidase
HAMD: Hamilton Depression Rating Scale
HDL: High-density Lipoprotein
LDL: Low-density Lipoprotein
LEfSe: Linear Discriminant Analysis Effect Size
MDD: Major Depressive Disorder
NIHSS: National Institutes of Health Stroke Scale
OPLS-DA: Orthogonal Projections on the Latent Structure Discrimination Analysis
PCA: Principal Component Analysis
PDW: Platelet Distribution Width
PO-PC: 1-palmitoyl-2-oleoyl-sn-glycero-3-phosphocholine.
PSD: Post-stroke Depression
ROC: Receiver Operating Characteristic Curves
SD: Standard Deviation
TIC: Total Ionic Strength Normalization
TMAO: Trimethylamine N-oxide
VIP: Variable Importance of Projection

## STANDARDS OF REPORTING

STROBE guidelines were followed.
