# Supplementary material for: The Role of Gut Bacteria and Plasma Metabolites in Predicting Post-Stroke Depression in Patients with Acute Ischemic Stroke
Source: Curr Neuropharmacol. 2025 Sep 9;24(10):1624–35. doi: 10.2174/011570159X390349250818115524 (PMC13647685; doi:10.2174/011570159X390349250818115524)
Supplement: Supplementary file 1 [file CN-24-10-1624_SD1.pdf]

Supplementary Material

The Role of Gut Bacteria and Plasma Metabolites in Predicting Post-Stroke Depression in Patients with Acute Ischemic Stroke

Lulu Wen[1,#], Tong Si[2,#], Chuming Yan[1], Huixin Shen[1], Wancheng Zheng[1], Meihong Xiu[3,*] and Miao Qu[1,*]

[1]Neurology Department, Xuan Wu Hospital Capital Medical University, Beijing, China; [2]Neurology Department, Beijing Shijitan Hospital, Beijing, China; [3]Beijing Huilongguan Hospital, Peking University Huilongguan Clinical Medical School, Beijing, China

SUPPLEMENTARY ONLINE CONTENT

eAppendix. Additional Methods

1.1 Clinical Data Acquisition and Plasma and Fecal Sample Preparation

All clinical data were obtained from the inpatient electronic medical record system, including demographic information, medical history, empirical results, imaging findings, and NIHSS scores. Blood samples were collected and centrifuged (1300 rpm, 10 minutes), and then the upper plasma was subpackaged and frozen at −80°C. Feces were initially collected using fecal specimen preservation tubes (AJcore, NA-012-T03, Ningbo, China), then subpackaged in cryopreservation tubes and maintained at −80°C.

1.2 16s RNA Sequencing

DNA from fecal samples was extracted as previously described[16]. 16S/18S rRNA gene was amplified and PCR products were quantified and qualified. The PCR product was then mixed and purified by the GeneJET Gel Extraction Kit. Subsequently, the Illumina TruSeq DNA PCR-Free Library preparation kit (Illumina, USA) was used to generate a sequencing library. Library quality was assessed by Qubit @ 2.0 fluorometer and Agilent bioanalyzer 2100 system. Lastly, the library was sequenced on the Illumina Nova Seq platform to generate 250 bp double-ended reads.

1.3 Sequencing Data Analysis

Paired-end reads from the original DNA fragments were merged using Fast Length Adjustment of Short (FLASH) reads, which were then assigned to each sample based on a unique barcode. Sequences were analyzed using the QIIME software package. First, reads were filtered through the QIIME quality filters, and sequences with ≥97 % similarity were assigned to identical OTUs. The representative sequences from each OTU were picked and annotated with taxonomic information using the RDP classifier. Alpha diversity and beta diversity were calculated to represent species diversity and microbial community composition, respectively. Finally, the linear discriminant analysis effect size (LEfSe) was used to screen the discriminant classification units of intestinal bacteria with significant differences between the PSD group and the non-PSD group.

1875-6190/26 $65.00+.00 © 2026 Bentham Science Publishers

1.4 Liquid Chromatography-Mass Spectrometry (LC-MS) for Untargeted Metabolome Analysis

In brief, 100µl plasma from each sample was augmented with 300µl ice-cold acetonitrile. The mixture was vortexed for 10 min at room temperature and incubated for 1 hour at - 20°C. After centrifuging at 14000g for 15 min at 4°C, the supernatant was transferred to the Accucore HILIC column (100×2.1 mm, 2.6 µm) at a flow rate of 0.3 mL/min using a 20-min linear gradient. The elution solutions for negative polarity mode were A (95% ACN, 10 mM ammonium acetate, pH 9.0) and B (50% ACN, 10 mM ammonium acetate, pH 9.0), while the elution solutions for positive polarity mode were C (0.1% FA in 95% ACN, 10 mM ammonium acetate) and D (0.1% FA in 95% ACN, 10 mM ammonium acetate). The solvent gradient was set as follows: 2% B/D, 1 min; 2-50% B/D, 16.5 min; 50-2% B/D, 2.5 min. Lastly, the Q-Exactive HF-X mass spectrometer (AB-SCIEX, USA) was operated in positive/negative polarity mode with a spray voltage of 3.2 kV and a capillary temperature of 320°C.

Table S1. Significant metabolites at baseline between PSD and non-PSD group.

Class	MS2 Name	rt	mz	FC	VIP	*P* value
Steroids and steroid derivatives	Progesterone	212.68	315.24	1.3	1.2	0.0067
Steroids and steroid derivatives	Dolichosterone	95.3	463.34	1.41	1.26	0.0038
Steroids and steroid derivatives	Secasterone	165.42	447.35	1.15	1.11	0.0205
Steroids and steroid derivatives	Chenodeoxycholic acid	113.15	391.28	1.24	1.17	0.0097
Steroids and steroid derivatives	5a-Tetrahydrocorticosterone	168.81	349.24	1.36	1.18	0.0429
Steroids and steroid derivatives	Cholic acid	168.82	407.28	1.24	1.13	0.0138
Glycerophospholipids	PC(P-18:1(9Z)/18:0)	145.77	772.62	1.24	1.2	0.0319
Glycerophospholipids	PC(16:1(9Z)/P-18:1(11Z))	39.56	742.58	1.23	1.21	0.0116
Glycerophospholipids	PC(18:1(9Z)/P-18:1(11Z))	37.6	770.6	1.11	1.14	0.037
Fatty Acyls	13S-hydroxyoctadecadienoic acid	96.05	279.23	1.29	1.23	0.0158
Fatty Acyls	Decanoylcarnitine	210.67	316.25	1.53	1.47	0.037
Fatty Acyls	Caprylic acid	51.7	143.11	1.18	1.13	0.018
Prenol lipids	Barringtogenol C	118.93	491.37	1.18	1.09	0.0495
Prenol lipids	Ganoderiol C	128.37	519.41	1.3	1.22	0.0147
Prenol lipids	22-Acetylpriverogenin B	96.69	517.39	1.25	1.16	0.0026
Prenol lipids	Camelliagenin A	96.01	475.38	1.52	1.31	0.0062
Organonitrogen compounds	Trimethylamine N-oxide	341.86	76.08	1.16	1.24	0.0468
N	Alpha-N-Phenylacetyl-L-glutamine	239.49	265.12	1.54	1.37	0.0309
Diazines	2-Ethyl-3,5-dimethylpyrazine	555.43	137.11	1.17	1.14	0.0036
Piperidines	2-Methylpiperidine	564.05	100.11	1.15	1.13	0.0169
Benzene and substituted derivatives	3-(4-Hydroxyphenyl)-1-propanol	31.62	153.09	1.16	1.1	0.0393
Organooxygen compounds	4-Aminobutyraldehyde	346.89	88.09	1.18	1.09	0.0024
Phenol ethers	1,1'-[1,11-Undecanediylbis(oxy)]bisbenzene	205.55	341.25	1.24	1.1	0.0393
Organooxygen compounds	2,2,6,7-Tetramethylbicyclo[4.3.0]nona-1(9),4-diene-7,8-diol	30.55	209.15	1.22	1.2	0.0265
Glycerolipids	DG(15:0/18:3(6Z,9Z,12Z)/0:0)	97.32	577.48	1.35	1.3	0.0468
Hydroxy acids and derivatives	Glycolic acid	195.52	75.01	1.07	1.06	0.0329
Phenols	3-Methoxy-4-hydroxyphenylglycol sulfate	42.99	263.02	1.42	1.22	0.0124
Phenols	[12]-Gingerol	168.42	377.27	1.39	1.18	0.0257
Steroids and steroid derivatives	Cholesterol sulfate	23.53	465.3	0.87	1.02	0.0417
Glycerophospholipids	PC(22:2(13Z,16Z)/14:1(9Z))	69.84	784.58	0.84	1.03	0.0212
Naphthalenes	1-nitrosonaphthalene	48.02	158.06	0.92	1.01	0.036
